# Reliability and Validity of the Turkish Version of the PedsQL 3.0 Cancer Module for 2- to 7-Year-Old and the PedsQL 4.0 Generic Core Scales for 5- to 7-Year-Old: The Hacettepe University Experience

**DOI:** 10.4274/tjh.2015.0242

**Published:** 2016-08-19

**Authors:** Vesile Yıldız Kabak, Yavuz Yakut, Mualla Çetin, Tülin Düger

**Affiliations:** 1 Hacettepe University Faculty of Health Sciences, Department of Physical Therapy and Rehabilitation, Ankara, Turkey; 2 Hacettepe University Faculty of Medicine, Department of Pediatrics, Division of Pediatric Hematology, Ankara, Turkey

**Keywords:** Pediatric Quality of Life Inventory, Validity, Reliability, children, Cancer

## Abstract

**Objective:**

The aim of this study was to investigate the reliability and validity of the Turkish version of the Pediatric Quality of Life Inventory (PedsQL) 3.0 Cancer Module for 2- to 7-year-old and the PedsQL 4.0 Generic Core Scales for 5- to 7-year-old in childhood cancer.

**Materials and Methods:**

The PedsQL 3.0 Cancer Module and PedsQL 4.0 Generic Core Scales were administered to children with cancer and their parents at Hacettepe University. Internal consistency was determined by using Cronbach’s alpha and test-retest reliability was determined by using the intraclass correlation coefficient (ICC). Construct validity was assessed by comparing the results of the PedsQL 3.0 Cancer Module with those of the PedsQL 4.0 Generic Core Scales.

**Results:**

Cronbach’s alpha of the PedsQL 3.0 Cancer Module varied from 0.803 to 0.873 and that of the PedsQL 4.0 Generic Core Scales from 0.665 to 0.841. Test-retest ICC values of the PedsQL 3.0 Cancer Module varied from 0.877 to 0.949 and those of the PedsQL 4.0 Generic Core Scales from 0.681 to 0.824. The correlation of the PedsQL 3.0 Cancer Module with subscale scores of the PedsQL 4.0 Generic Core Scales showed that there were excellent to fair correlations between the two scales. The relationship between parent proxy-report and child self-report of the PedsQL 3.0 Cancer Module had very good correlation (r=0.694, p<0.001), as did the PedsQL 4.0 Generic Core Scales (r=0.540, p=0.002).

**Conclusion:**

This study demonstrated the reliability, validity, and feasibility of the Turkish version of the PedsQL 3.0 Cancer Module in 2- to 4-year-old and 5- to 7-year-old and the PedsQL 4.0 Generic Core Scales in 5- to 7-year-old in childhood cancer.

## Abstract (tr)

**Amaç:**

Çalışmamızın amacı, çocukluk çağı kanserlerinde Çocuklar için Yaşam Kalitesi Ölçeği (ÇİYKÖ) 3.0 Kanser Modülü Türkçe versiyonunun 2-7 yaşları arasındakiler için ve ÇİYKÖ 4.0 Genel Skalası’nın 5-7 yaşları arasındakiler için geçerliliğini ve güvenilirliğini araştırmaktır.

**Gereç ve Yöntemler:**

ÇİYKÖ 3.0 Kanser Modülü ve ÇİYKÖ 4.0 Genel Skalası Hacettepe Üniversitesi’ndeki kanserli çocuklara ve ebeveynlerine uygulanmıştır. Cronbach’s alpha kullanılarak iç tutarlılık, sınıf içi korelasyon katsayısı (SKK) kullanılarak test-retest güvenirliği belirlenmiştir. Yapı geçerliliği ÇİYKÖ 3.0 Kanser Modülü ile ÇİYKÖ 4.0 Genel Skalası sonuçlarının karşılaştırılmasıyla değerlendirilmiştir.

**Bulgular:**

ÇİYKÖ 3.0 Kanser Modülü’nün Cronbach’s alpha değerleri 0,803-0,873 arasında, ÇİYKÖ 4.0 Genel Skalası’nın ise 0,665-0,841 arasında değişiklik göstermektedir. Test-retest SKK değerleri ise ÇİYKÖ 3.0 Kanser Modülü’nde 0,877-0,949 arasında, ÇİYKÖ 4.0 Genel Skalası’nda ise 0,681-0,824 arasında değişiklik göstermektedir. ÇİYKÖ Kanser Modülü ile Genel Skalası’nın alt başlık skorlarının korelasyonlarının mükemmel-orta düzeyde olduğu gösterilmiştir. ÇİYKÖ Kanser Modülü Çocuk Formu ile Ebeveyn Formu arasındaki ilişki çok iyi düzeyde korele (r=0,694, p<0,001), ÇİYKÖ Genel Skalası’nda ise iyi düzeyde koreledir (r=0,540, p=0,002).

**Sonuç:**

Bu çalışma ÇİYKÖ 3.0 Kanser Modülü’nün Türkçe versiyonunun 2-4 yaş ile 5-7 yaşları arasındaki ve ÇİYKÖ 4.0 Genel Skalası Türkçe versiyonunun 5-7 yaşları arasındaki kanserli çocuklarda geçerli, güvenilir ve uygulanabilir olduğunu göstermiştir.

## INTRODUCTION

Quality of life (QOL) has been described as a subjective term and is defined as a person’s sense of social, emotional, and physical well-being and his/her ability to function in ordinary tasks of daily living [1,2,3,4]. Therefore, a health-related quality of life (HRQOL) instrument should include physical, mental, and social health dimensions [5,6]. It is increasingly acknowledged as an important health outcome measure in clinical trials and health service research and evaluation [7].

Disease-specific HRQOL assessment instruments have been developed to determine the impact of disease and treatment on the quality of patients’ life. However, there are a limited number of instruments designed to measure the HRQOL of pediatric patients with cancer [8,9,10].

The Pediatric Quality of Life Inventory (PedsQL), which has both generic and disease-specific modules as well as patient and parent versions, is one of the very few instruments that is widely used to assess HRQOL among children and adolescents between the ages of 2 and 18 years [3,11]. It is brief, can be applied in 5-15 min, and can be scored easily [12,13]. Evaluation is conducted by both children and parents; children aged 5 to 18 years are asked to evaluate their own HRQOL (child self-report) and the parents of children aged 2 to 18 years are asked to evaluate their child’s HRQOL (parent proxy-report) [14]. The PedsQL 4.0 Generic Core Scales were specifically designed for application in both healthy and patient populations. The PedsQL 3.0 Cancer Module was designed to measure HRQOL dimensions specific to pediatric cancers [7]. This instrument has already been validated in English [15], German [16], Chinese [11], Japanese [14], Urdu [17], and Portuguese [8]. In Turkey, the validity and reliability of the PedsQL 4.0 Generic Core Scales for 8- to 12-year-old and 13- to 18-year-old children were evaluated, as was the PedsQL 3.0 Cancer Module for 8- to 12-year-old children [18,19,20]. Uneri et al. investigated the reliability and validity of the Turkish translation of the PedsQL 4.0 Generic Core Scales for 2- to 4-year-old and 5- to 7-year-old Turkish children. They found that the validity of the parent proxy-reports for the two age groups was sufficient, whereas the validity of the child self-report of the 5- to 7-year-old age group was low [12].

The aim of this study was to investigate the reliability and validity of the Turkish version of the PedsQL 3.0 Cancer Module for 2- to 4-year-old and 5- to 7-year olds and the reliability and validity of the Turkish version of the PedsQL 4.0 Generic Core Scales for 5- to 7-year-old in childhood cancer.

## MATERIALS AND METHODS

### Patients and Setting

This study was developed in Turkey. We recruited children with cancer and their parents from Hacettepe University Hospital. Children between the ages of 2 and 7 who were diagnosed with cancer at least 2 months earlier, who agreed to participate in the study, and who had good verbal communication were included. Children with comorbid disease, major developmental disorders, and neurologic problems were excluded from the study. The 2- to 4-year-old age group consisted of 43 children and their parents. The 5- to 7-year-old age group consisted of 31 children and their parents. Informed consent was obtained from all individual participants included in the study.

### Instruments

#### PedsQL 4.0 Generic Core Scales

The PedsQL 4.0 Generic Core Scales were designed by Varni et al. in 1999 as a HRQOL measurement for children and adolescents aged between 2 and 18 years [13]. It consists of 23 items: physical functioning (8 items), emotional functioning (5 items), social functioning (5 items), and school functioning (5 items). Child self-reports include 3 age groups: 5-7 years (young children), 8-12 years (children), and 13-18 years (teens). The parent proxy-report, however, includes 4 age groups: 2-4 (toddlers), 5-7, 8-12, and 13-18. The response scale is a 5-point Likert scale for all age groups, except for the 5- to 7-year-old’ version. Items are reverse-scored and linearly transformed to a 0-100 scale, so that higher scores indicate better HRQOL. The response scale of the 5- to 7-year-old version is completed with the help of an interviewer and simplified to a 3-point scale (0, 2, and 4 points). The child answers the items with the help of a visual scale (happy, neutral, and sad faces). The PedsQL 4.0 computes the scale scores as well as the Psychosocial Health Summary Scores by adding the sum of points from the Emotional, Social, and School Functioning Subscales and dividing them by the total number of items answered [21,22].

#### PedsQL 3.0 Cancer Module

The PedsQL 3.0 Cancer Module was developed and tested for validity and reliability by Varni et al. [3,22,23]. This module consists of 27 items: pain and hurt (2 items), nausea (5 items), procedural anxiety (3 items), treatment anxiety (3 items), worry (3 items), cognitive problems (5 items), perceived physical appearance (3 items), and communication (3 items). The format, instructions, Likert response options, and scoring method are similar to those of the PedsQL 4.0 Generic Core Scales [22]. This scale comprises two parallel forms for child and for parent. Higher scores indicate better HRQOL [21].

### Translation and Cross-Cultural Adaptation

This study was conducted in these phases:

1. Translation, validation, and reliability of the PedsQL 3.0 Cancer Module for 2- to 4-year-old parent proxy-report and 5- to 7-year-old children’s form and parent proxy-report.

2. Translation, validation, and reliability of the PedsQL 4.0 Generic Core Scales for 5- to 7-year-old children’s form.

In the translation process, we used the guidelines for cross-cultural adaptation and we obtained permission for the Turkish version from Varni et al. (Mapi Research Trust) [4,24]. Approval of the study was obtained from the Ethics Committee of Hacettepe University (GO 14/455).

The original English instruments (PedsQL 4.0 Generic Core Scale /5-7 years and PedsQL 3.0 Cancer Module/2-4 and 5-7 years) were translated independently into Turkish. Two translations from English to Turkish were done by two different and independent native Turkish translators. The Turkish translations were then compared for inconsistencies. The two translations were then retranslated, also blindly and independently, into English by two native English speakers. The Turkish version was then jointly reviewed by a bilingual team, including the four translators, three physical therapists, and a physician, to assess the necessity of cultural adaptation. The Turkish version was compared with the original English version to detect possible errors of interpretation and nuances that might have been missed. The final stage of the adaptation process was to test the prefinal version. Ten children were tested in this stage. The results eliminated the necessity for Turkish cultural adaptation.

## Statistical Analysis

### Reliability

Two common forms of reliability are test-retest reliability and internal consistency. For test-retest reliability, the forms were applied in 7-day intervals. We used the intraclass correlation coefficient (ICC) to evaluate test-retest reliability. The ICC can vary from 0.00 to 1.00, where values of 0.60 to 0.80 are regarded as evidence of good reliability and those above 0.80 indicate excellent reliability [25,26,27].

The internal consistency of a scale relates to its homogeneity. The coefficient of internal consistency is mainly assessed with Cronbach’s alpha. It is suggested that the value of alpha should be above 0.80 for acceptance as high internal consistency [28].

### Validity

In this study, construct validity was assessed by comparing the responses to the PedsQL 3.0 Cancer Module to the results of the PedsQL 4.0 Generic Core Scales. Construct validity coefficients (r) were accepted as follows: 0.81-1.0 as excellent, 0.61-0.80 very good, 0.41-0.60 good, 0.21-0.40 fair, and 0-0.20 poor. Construct validity was measured by Pearson’s correlation coefficient [29].

All assessments were repeated 7 days later by the physical therapist. Means and standard deviations were determined to describe the demographic data of the patients. All statistical analyses were done with IBM-SPSS 22.0 for Windows. A probability value of p<0.05 was considered to indicate a significant effect.

## RESULTS

### Demographic Characteristics

The total sample consisted of 74 children with cancer, aged 2 to 7 years old, and their parents. While 60% of the children had leukemia, the others had different cancers (lymphoma, brain tumors, rhabdomyosarcoma, Wilms’ tumor, osteosarcoma, and Ewing’s sarcoma). The number of children in the 2- to 4-year-old group was 43 (58.10%) and in the 5- to 7-year-old group was 31 (41.89%). Characteristics of the groups are summarized in Table 1. Subscale and total scores of the 2- to 4-year-old and 5- to 7-year-old groups are summarized in Table 2.

### Reliability

#### Internal Consistency Reliability

The internal consistency reliability alpha coefficient (Cronbach’s coefficient alpha) of the PedsQL 3.0 Cancer Module for the 2- to 4-year-old parents’ form was 0.803, and those for the 5- to 7-year-old parents’ and children’s forms were 0.867 and 0.817, respectively. Cronbach’s coefficient alpha values of the PedsQL 4.0 Generic Core Scales 5- to 7-year-old total score were 0.704 for the parents’ form and 0.712 for the children’s form.

#### Test-Retest Reliability

For the 2- to 4-year-old parents’ form, the test-retest ICC value of the PedsQL 3.0 Cancer Module total score was 0.877 [95% confidence interval (CI)=0.774-0.934]. For the 5- to 7-year-old, ICC values of the parents’ and children’s forms were 0.949 (95% CI=0.898-0.975) and 0.889 (95% CI=0.780-0.945), respectively. The ICC values of the PedsQL 4.0 Generic Core Scales total scores for parents’ and children’s forms were 0.824 (95% CI=0.599-0.928) and 0.681 (95% CI=0.425-0.837), respectively.

#### Total Subscale Correlation

Good and very good correlations were found between total scores of the scales and the subscale scores, as shown in Table 3.

### Validity

#### Construct Validity

Examination of the correlation of the 2- to 4-year-old parent proxy-report of the PedsQL 3.0 Cancer Module with the subscale scores of the PedsQL 4.0 Generic Core Scales showed that there were excellent to fair correlations between the two scales, except for the subscales of ‘worry’ and ‘school-related problems’. Table 4 indicates the intercorrelations between the PedsQL 3.0 Cancer Module and PedsQL 4.0 Generic Core Scales for the 2- to 4-year-old parent proxy-report.

Examination of the correlation of the 5- to 7-year-old parent proxy-report of the PedsQL 3.0 Cancer Module with the subscale scores of the PedsQL 4.0 Generic Core Scale showed that there were very good to fair correlations between the two scales. Table 5 indicates the intercorrelations between the PedsQL 3.0 Cancer Module and the PedsQL 4.0 Generic Core Scales for the 5- to 7-year-old parent proxy-report. Table 6 indicates the intercorrelation between the PedsQL 3.0 Cancer Module and PedsQL 4.0 Generic Core Scales for 5- to 7-year-old children’s self-reports.

When the relationship between parents’ and children’s forms for the 5- to 7-year-old PedsQL 3.0 Cancer Module was analyzed, a very good correlation was found (r=0.694, p<0.001). For the 5- to 7-year-old PedsQL 4.0 Generic Core Scales, a good correlation was found between parents’ and children’s forms, which was also statistically significant (r=0.540, p=0.002).

## DISCUSSION

This study demonstrated the reliability, validity, and feasibility of the Turkish version of the PedsQL 3.0 Cancer Module in 2- to 4-year-old and 5- to 7-year-old and the PedsQL 4.0 Generic Core Scales in 5- to 7-year-old with childhood cancers. The analyses support the reliability and validity of the instrument as a child self-report and parent proxy-report HRQOL measurement instrument for Turkish pediatric cancer patients. The PedsQL is brief and easy to complete.

The PedsQL self-report and proxy-report internal consistency reliabilities generally exceeded the recommended minimum alpha coefficient standard of 0.70 for group comparisons. Across the ages, the PedsQL 4.0 Generic Core Scales Total Score for both child self-report and parent proxy-report approached or exceeded an alpha of 0.90, recommended for individual patient analysis, making the Total Scale Score suitable as a summary score for the primary analysis of HRQOL outcome in clinical trials and other group comparisons [4].

Uneri et al. investigated the reliability and validity of the Turkish version of the PedsQL 4.0 Generic Core Scales for 5- to 7-year-old Turkish children. They found that internal consistency reliability alpha coefficients (Cronbach’s coefficient alpha) of the total scale score for parent-proxy report and for the child’s self-report of the 5- to 7-year-old age group were 0.86 and 0.80. The validity of the parent-proxy report was found to be sufficient, whereas the validity of the child self-report was low. When the concordance between the child self-report and parent-proxy reports was analyzed, statistically significant but low correlations were found between the total scores [12]. The reason for this situation, similar to our study, may be the low number of patients included. This can be considered as a limitation of our study.

In our study, we found that the Cronbach’s coefficient alpha values of the PedsQL 4.0 Generic Core Scales total score varied from 0.665 to 0.841 for 5- to 7-year-old, test-retest ICC values were 0.681-0.824 (good), and the correlations between total score and subscale scores were good to excellent. Eiser and Morse stated that, in practice, values greater than 0.50 might be considered as acceptable in chronic disease of childhood QOL measurements [30]. Thus, the reliability of the PedsQL 4.0 Generic Core Scales for 5- to 7-year-old may be considered as acceptable.

For the PedsQL 4.0 Generic Core Scales the correlation between parent proxy-report and child self-report of 5- to 7-year-old was significant and good (r=0.540, p=0.002). This lower correlation and internal consistency of the 5- to 7-year-old age group may be related to school functioning. In Turkey, children of this age do not go to school yet, and children with chronic diseases quit school. Therefore, most of the children in this group had difficulties understanding school functioning questions and some parents mentioned that their children were too young to understand some of the questions. Only 16 parents and children answered the school functioning questions, which might be due to misunderstanding some questions (for example, forgetting things). This indicated that not only are some modifications to the school functioning questions for children aged 5-7 years necessary, but also that the parent-proxy report and child self-report should be applied together in this age group.

The PedsQL 3.0 Cancer Module Scales internal consistency reliabilities generally exceeded the recommended minimum alpha coefficient standard of 0.70 for group comparison for child self-report for ages 8-18 years and parent proxy-report for ages 2-18 years. For self-report at ages 5-7 years, only the procedural anxiety and treatment anxiety scales met the 0.70 standard and for most scales values were in the range of 0.80 to 0.90 [4].

In our study, Cronbach’s coefficient alpha of the PedsQL 3.0 Cancer Module for 2- to 7-year-old was from 0.803 to 0.73 for the parent-proxy reports and the child self-reports (high). Test-retest ICC values were 0.877 to 0.949 (excellent) and the correlations between total score and subscale scores for 5- to 7-year-old were higher than those for 2- to 4-year-old. The Turkish version of the PedsQL 3.0 Cancer Module in children aged 2-7 years can be applied for the Turkish population.

The intercorrelations between the PedsQL 3.0 Cancer Module and PedsQL 4.0 Generic Core Scales parent proxy-reports were in the range of moderate to high, except for ‘worry’ and ‘school-related problems’ in 2- to 4-year-old, and except for ‘pain and hurt’ and ‘physical functioning’ in 5- to 7-year-old. In Turkey, many children between 2 and 4 years old do not go to school or kindergarten. Therefore, not all parents answered schooling questions. At these ages, on the other hand, children do not know about their disease and its treatment. In 5- to 7-year-old, subscales and their questions of the cancer module do not correlate with physical situations. According to the results of 5- to 7-year-old, while parents correlated physical dysfunctions with ‘pain and hurt’, children correlated them with ‘nausea’. In this self-report, ‘procedural anxiety’ and ‘perceived physical appearance’ do not correlate with Generic Core Scale subscales, and the ‘social functioning subscale’ of the Generic Core Scale does not correlate with Cancer Module subscales. These low correlations in subscales might be due to the small number of items that compose the subscales, the low level of schooling in these ages, or the absence of physical function subscales in the cancer module. Total scale score may be suitable as a summary score for the primary analysis of the PedsQL Inventory in clinical trials and other group comparisons.

Parent-child agreement about QOL is controversial in the literature [30]. Some studies reported high agreement, whereas others reported low agreement [31,32,33]. In our study, there was an excellent correlation between the parent-proxy report and child self-report for 5- to 7-year-old with the PedsQL 3.0 Cancer Module. Although patient self-report is considered the standard for measuring perceived HRQOL, it is the parent’s perception of the child’s HRQOL that may influence healthcare utilization [34]. In clinical practice, there may be circumstances in which the child is too young to understand the questions, or too ill and unwilling to complete an instrument. Therefore, in cases in which pediatric patients are not able to provide self-reports, reliable and valid parent proxy-report instruments are needed [35].

In conclusion, our results indicated that the Turkish versions of the PedsQL 4.0 Generic Core Scales for 5- to 7-year-old and the PedsQL 3.0 Cancer Module for 2- to 7-year-old are easy to understand, reliable, and valid instruments in the Turkish-speaking population. These instruments may be utilized as outcome measures in pediatric cancer clinical trials, research, and clinical practice for HRQOL outcome assessment. However, we suggest that the parent proxy-report and child self-report should be used together for 5- to 7-year-old. Further studies should focus on testing the responsiveness and reliability of the PedsQL in patients who continue or finish treatments and in long-term follow-up measurements.

## Ethics

Ethics Committee Approval: The approval of the Ethic Committee of the Hacettepe University was obtained about this study (GO 14/455); Informed Consent: It was taken.

## Figures and Tables

**Table 1:**
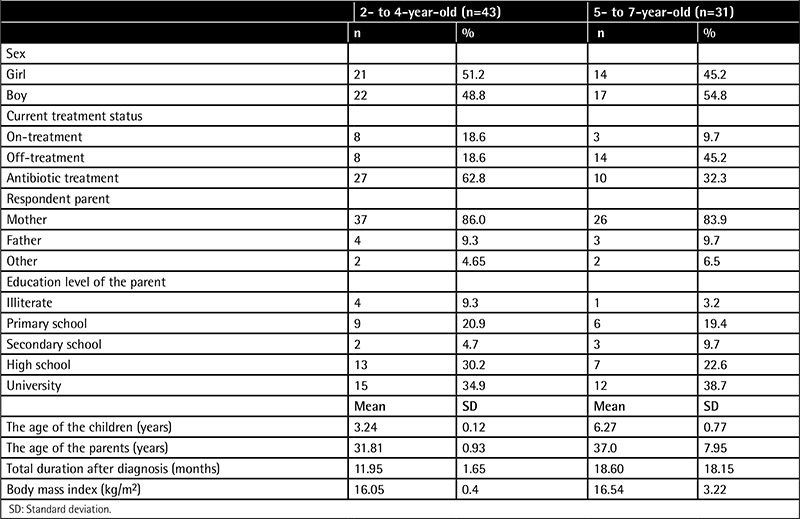
Demographic characteristics of the samples.

**Table 2:**
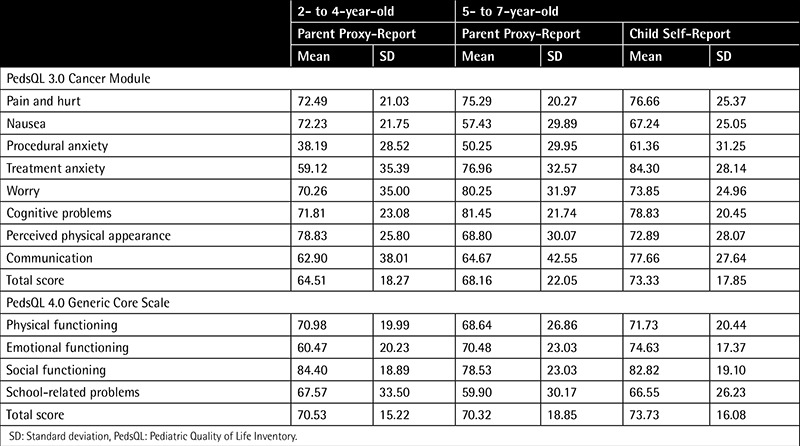
Pediatric Quality of Life Inventory 3.0 Cancer Module and 4.0 Generic Core Scale Scores.

**Table 3:**
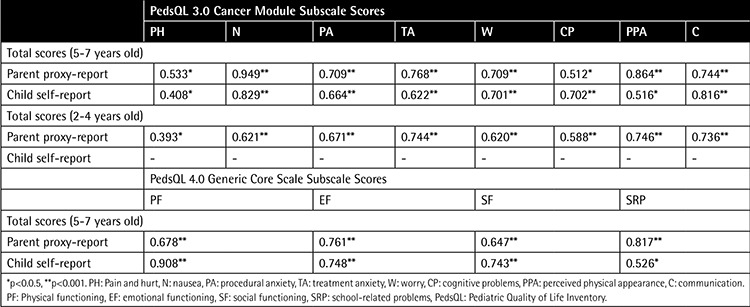
Pearson’s correlation coefficients between total scores and subscale scores of the scales.

**Table 4:**
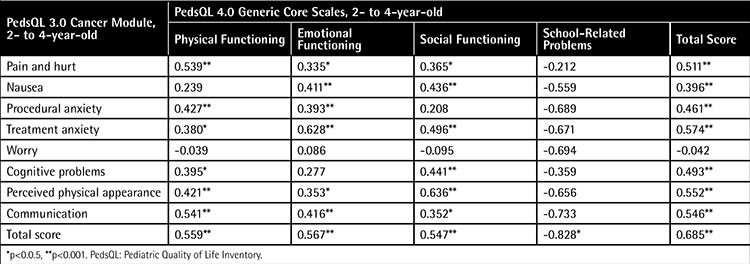
Pearson’s correlation coefficients between subscale scores of the 2- to 4-year-old parent proxy-report of the Pediatric Quality of Life Inventory 3.0 Cancer Module and Pediatric Quality of Life Inventory 4.0 Generic Core Scales.

**Table 5:**
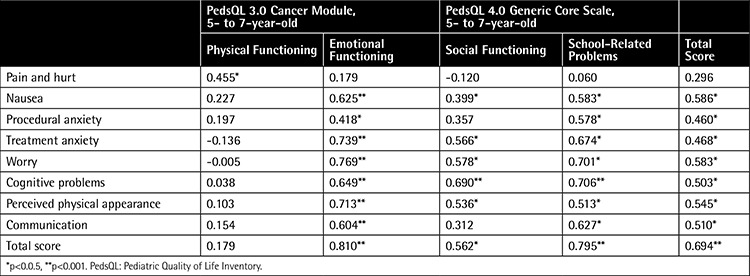
Pearson’s correlation coefficients between subscale scores of the 5- to 7-year-old parent proxy-report of the Pediatric Quality of Life Inventory 3.0 Cancer Module and Pediatric Quality of Life Inventory 4.0 Generic Core Scales.

**Table 6:**
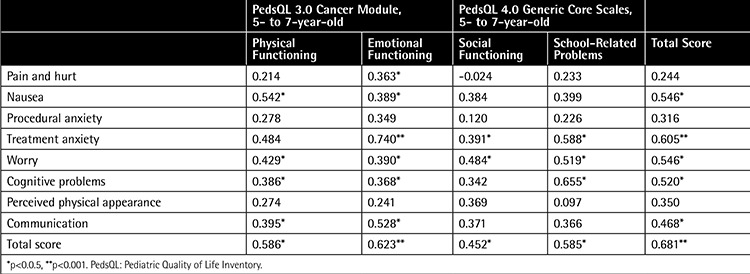
Pearson’s correlation coefficients between subscale scores of the 5-to 7- year old child self-report of the Pediatric Quality of Life Inventory 3.0 Cancer Module and Pediatric Quality of Life Inventory 4.0 Generic Core Scales.
